# Feline panleukopenia virus and canine parvovirus: development of two qPCR assays with high-resolution melting analysis and molecular epidemiology in dogs and cats from Northen Italy in 2017–2023

**DOI:** 10.3389/fvets.2026.1789452

**Published:** 2026-03-04

**Authors:** Andrea Balboni, Veronica Facile, Lorenza Urbani, Martina Magliocca, Laura Gallina, Maddalena Vigato, Mara Battilani

**Affiliations:** Department of Veterinary Medical Science, Alma Mater Studiorum – University of Bologna, Bologna, Italy

**Keywords:** companion animal, genetic characterization, molecular detection, protoparvovirus, VP2

## Abstract

The *Protoparvovirus carnivoran* 1 viral species includes relevant pathogens as feline panleukopenia virus (FPV) and canine parvovirus type 2 (CPV-2), which are mainly responsible for immunosuppression and gastroenteritis in domestic and wild carnivores. Differently to FPV, CPV is frequently subjected to mutations. To date, the original antigenic type CPV-2 is mainly used in vaccine production, while field strains have been progressively replaced by the CPV antigenic variants 2a, 2b, and 2c. In recent years, additional distinctive mutations have been identified in different CPV antigenic variants classified as “Asian-like.” The variability of these viruses can impact on the reliability of molecular diagnostic tests potentially leading to false-negative results or delays in diagnosis. To improve diagnostic accuracy and efficiency, innovative molecular techniques such as High-Resolution Melting (HRM) analysis have been developed. These methods reduce execution time, facilitate diagnosis, and enable the differentiation of species or variants without the need for sequencing. In this study, two real-time PCR (qPCR) assays with HRM analysis were developed to complement existing tools for the detection and genetic differentiation of circulating FPV and CPV. Specifically, a test capable of differentiating FPV, original CPV-2 and CPV-2 antigenic variants, and a test for the identification of Asian-like CPV strains were validated. Furthermore, the FPV and CPV-2 identified in 33 dogs and cats diagnosed with parvoviral infection in a veterinary teaching hospital in Northern Italy between 2017 and 2023 were genetically characterized by sequencing. Based on specific VP2 amino acid residues, 33.3% viruses were FPV, 6.1% were original CPV-2, 6.1% were CPV-2a, 21.2% were CPV-2b and 33.3% were CPV-2c. FPV were detected only in cats and showed high amino acid similarity, confirming its evolutionary stasis. The 45.4% of CPV identified in this study, carried amino acid residues resembling those of Asian-like viruses, suggesting an origin linked to an initial importation and subsequent local diffusion. In contrast, the other CPV-2a, 2b, and 2c viruses exhibited greater genetic heterogeneity and their autochthonous origin was supposed. The two qPCR-HRM assays successfully detected and classified all the FPV and CPV tested, highlighting their reliability and usefulness for both diagnostic and epidemiological purposes.

## 1 Introduction

The *Protoparvovirus carnivoran 1* is a recently defined viral species that includes both Feline panleukopenia virus (FPV) and Canine parvovirus type 2 (CPV-2 or more commonly CPV) which have significant relevance for the health and management of both domestic and wild carnivores (1, 2). Infections with these viruses in dogs and cats are associated with severe and life-threating clinical signs and can frequently be fatal, especially in young subjects (3, 4). They are small non-enveloped linear ssDNA viruses, members of the family Parvoviridae and genus Protoparvovirus. Since first identification, their genome has undergone continuous mutation (5, 6). The genome of these viruses is composed of two genes: REP which encodes non-structural protein (NS1 and NS2) and CAP which encodes for structural protein (VP1, VP2, and VP3). VP2 is the major structural protein, which induces the production of protective antibodies and is responsible for host adaptability, pathogenicity, and virulence of the virus (7, 8). FPV was the first parvovirus described in companion animals (9) and it is well established that CPV originated from FPV-like viruses in wild animal species, after mutations in the VP2 protein that allow changing in hosts range (10, 11). Specifically, amino acid substitution at position 93, 103 and 323 of VP2 protein allowed CPV binding with transferrin receptor (TfR) and replication in canine host (12, 13); whereas amino acid substitution in position 80, 564 and 568 resulted in the impossibility of replicating in feline host (14, 15). The original CPV-2 strain identified in 1970 is now rarely detected in natural infections and is mainly used in vaccine production (16). Differently to FPV which shows genetic stability (5), CPV is frequently subjected to mutations and in few years the so-called CPV-2 variants 2a, 2b and 2c emerged and have become the predominant viruses causing disease in domestic dogs (17, 18). The most evolutionarily significant amino acid substitutions in VP2 protein (Met87Leu, Ala300Gly, and Asp305Tyr) enabled these variants to regain affinity for the TfR, thereby expanding its host range to include feline populations and cause clinical signs comparable to FPV infection (19). Furthermore, these three antigenic variants are characterized by distinct amino acid substitutions in the VP2 protein that differentiate each other. CPV-2a, which originated directly from the original CPV-2 in 1980 (20), differs from CPV-2 by three additional amino acid substitutions, notably Ile101Thr, Ser297Ala, and Val555Ile (19, 21, 22). CPV-2b, reported in 1984, evolved from CPV-2a due to a single amino acid change in position 426 (Asn426Asp) (23), and it also retains Ser297Ala and Ile555Val (24). Finally, CPV-2c was reported from 2000 and emerged from CPV-2b via mutation Asp426Glu (25). More recently, additional distinctive mutations have been identified in different CPV antigenic variants classified as “Asian-like,” as reported for the first time in Asia. These viruses have shown a progressive worldwide diffusion in dogs (26), cats (27) and wild animals (28) and are potentially responsible for vaccine failure (29). All the Asian-like CPV have the amino acid residues 267Tyr and 324Ile. In Asian-like CPV-2a and CPV-2b, the amino acid mutation Thr440Ala has also occasionally been reported. In contrast, in Asian-like CPV-2c, two additional amino acid substitutions, Ala5Gly and Gln370Arg, were identified and increasingly reported globally (30). The Asian-like CPV-2c appear to be the absolute dominant compared to the other Asian-like CPV variants in dogs in Asia, and it started spreading also in Europe and Africa (30–34). The emergence of genetic mutations potentially associated with viral tropism, fitness, virulence, antigenicity and vaccine efficacy, requires continuous surveillance on circulating FPV and CPV. Such genetic changes can also influence diagnostic accuracy, leading to false-negative results and errors in treatment and management strategies (35). For these reasons, diagnosis must be rapid, sensitive and specific. Molecular techniques have consistently proven to be the most effective methods for diagnosing parvoviral infection (36). The current gold standard for the diagnosis of FPV and CPV infection are molecular assays, such as end-point polymerase chain reaction (PCR) or real-time PCR (qPCR), which detect the viral DNA in biological matrices such as feces (37–39). Indeed, although they require expensive and sophisticated instrumentation, they offer unparalleled sensitivity and specificity compared to other approaches. Moreover, with the advent of qPCR, it is now possible to quantify the viral load, helping to adopt adequate prophylactic measures to prevent infection, particularly in kennels and shelters, where this virus is often responsible for severe epizootics (39, 40). Regarding epidemiological investigations and monitoring of viral evolution, sequencing of the complete VP2 gene or specific regions of it represents the most established method. Although indispensable for assessing individual nucleotide mutations, sequencing remains costly and time-consuming. In recent years, innovative molecular diagnostic techniques, such as loop-mediated isothermal amplification and High-Resolution Melting (HRM) analysis, have been developed to reduce execution time and facilitate the diagnosis (41, 42). HRM analysis, in particular, allows discrimination and differentiation between species, genotypes or variants without the need of sequencing, by detecting minimal melting temperature variation linked to differences in the target sequence of even a single nucleotide (43). To date two qPCR assays with HRM analysis have been developed: one for the discrimination of CPV from FPV and another for identifying CPV-2a, 2b, and 2c variants (44, 45). The first aim of the present study was to develop and validate two qPCR assays with HRM analysis to complement existing tools for the genetic differentiation of circulating FPV and CPV. Specifically, the first one was a test capable of differentiating FPV, original CPV-2 and CPV-2 antigenic variants (2a, 2b, and 2c). This discrimination is essential for the correct management of parvovirosis in dogs and cats, and avoid false positive results related to vaccine-strains. The second was a test for the identification of Asian-like CPV. As previously stated, viruses with these new distinctive mutations can be characterized to an improved fitness and lead to vaccine failure. For this reason, it is important to monitor their diffusion and promptly intervene in case of outbreaks. The second aim of this study was to use the developed assays, in association with nucleotide sequencing of the complete VP2 gene, to identify and genetically characterize circulating FPV and CPV strains in dogs and cats referred to a Veterinary Teaching Hospital (VTH) in Northen Italy from 2017 to 2023.

## 2 Materials and methods

### 2.1 Real-time PCR with high-resolution melting analysis

#### 2.1.1 Primer design

To design appropriate primers, sequences from 87 reference FPV and CPV strains were retrieved from the GenBank database^1^ and aligned using the ClustalW method implemented in BioEdit software version 7.7. To verify the specificity of the selected primers, a BLAST search^2^ was performed. The web interface Oligo Calc^3^ was used to exclude secondary structure and primer dimers formation.

**Figure 1:**
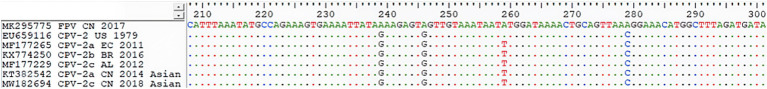
Nucleotide fragment amplified by the qPCR-HRM-1 assay (93 nts of the VP2 gene, position 208–300 of the GenBank reference sequence EU659116).

The first qPCR-HRM assay (qPCR-HRM-1) had the intent to differentiate FPV, original CPV-2 and CPV-2 antigenic variants (2a, 2b, and 2c). The assay targeted a fragment of 93 nucleotides (nts) between positions 208 and 300 of the VP2 gene (GenBank reference sequence EU659116) showing conserved nucleotide mutations between the different viruses (Figure 1). Specifically, four point mutations distinguished FPV and CPV (A-G at position 239 and 246; A-T at position 259; A-C at position 279) leading to four amino acid substitutions (80lysine to arginine, 87methionine to leucine, 93lysine to asparagine, and 103valine to alanine, respectively). In the same tract, at position 259, an A-T mutation differentiated original CPV-2 from CPV-2 antigenic variants (2a, 2b, and 2c), resulting in an amino acid change from 87methionine to leucine.

**Figure 2:**
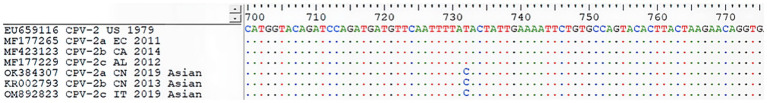
Nucleotide fragment amplified by the qPCR-HRM-2 assay (72 nts of the VP2 gene, position 703–774 of the GenBank reference sequence EU659116).

The second qPCR-HRM assay (qPCR-HRM-2), was aimed to be used only on CPV-positive samples to differentiate Asian-like CPV strains from other CPVs. The assay targeted a fragment of 72 nts between positions 703 and 774 of the VP2 gene (GenBank reference sequence EU659116) showing a single nucleotide mutation (T-C) between the differentiated viruses (Figure 2). This single nucleotide polymorphism (SNP), being a synonymous mutation, did not involve amino acid change (tyrosine at residue 244).

The set of primers designed and used in the present study are reported in Table 1.

**Table 1:** Primers designed and used in this study.

Molecular method	Primers	Fragment length	Target differentiation
qPCR-HRM-1	Parvo_VP2_208-229_for: CAT TTA AAT ATG CCA GAA AGT G Parvo_VP2_280-300_rev: ATC ATC TAA AGC CAT GTT TCC	93 nts	- FPV - original CPV-2 - CPV-2 antigenic variants (2a, 2b and 2c)
qPCR-HRM-2	Parvo_VP2_703-723_for: GGT ACA GAT CCA GAT GAT GTT Parvo_VP2_757-774_rev: TCA TCA CCT GTT CTT AGT AAG TG	72 nts	- Asian-like CPV - other CPVs
End-point PCR for plasmid insert	Parvo_VP2_208-229_for Parvo_VP2_757-774_rev	567 nts	

CPV, Canine parvovirus; FPV, Feline panleukopenia virus; nts, nucleotides; qPCR-HRM, Real-time PCR with High-Resolution Melting analysis.

#### 2.1.2 qPCR and HRM analysis

Reactions were performed using the QuantStudio 3 Real-Time PCR System (Thermo Fisher Scientific, Applied Biosystems, Waltham, MA, USA) with a total reaction volume of 50 μL per tube. Each tube contained 25 μL Applied Biosystems MeltDoctor reagents (Thermo Fisher Scientific, Life Technologies, Waltham, MA, USA), 3 μL of forward and reverse primers, respectively, with a concentration of 5 μM each, 2.5 μL of DNA template, and 16.5 μL of water for molecular biology. Each sample was tested in triplicate. The cycling conditions included a denaturation stage at 95 °C for 10 min, followed by the PCR stage of 40 cycles at 95 °C for 15 s and 60 °C for 1 min. The determination of melting curve was performed as follows: 95 °C for 10 s, 60 °C for 1 min to 95 °C for 15 s at a rate of 0.025 °C/s. HRM analysis was conducted using the Applied Biosystems High Resolution Melt Software v3.1 (Thermo Fisher Scientific, Waltham, MA, USA). The software automatically assigned samples to variants based on melting curve similarity with positive controls. This analysis was made possible by the use of specific saturating DNA dye, which maximizes the detection of mismatched duplexes, and an advanced qPCR system that enables rapid data acquisition (46). The base substitution A-T differentiating the original CPV-2 from CPV-2 antigenic variants (2a, 2b, and 2c) in the qPCR-HRM-1 assay represents a big challenge in SNP detection due to minimal difference in melting temperatures (< 0.2 °C) (47). For this reason, to be able to discriminate the two viral types, a further step was performed. Specifically, PCR product obtained from a CPV-2b positive sample was mixed with each amplicon of the unknown samples in a 1:1 (v/v) ratio to form heteroduplex. The mixed samples were denatured at 95 °C for 1 min and renatured at 30 °C for 2 min before HRM reanalysis (same protocol described above).

#### 2.1.3 Validation of the molecular assays

To validate the qPCR assays developed, sensibility and specificity were evaluated. Sensibility was assessed using ten-fold serial dilutions (ranging from 1 × 10^0^ to 1 × 10^7^ copies/μL) of a DNA recombinant plasmid containing a fragment of 567 nts in length of the VP2 gene (created with the forward primer of qPCR-HRM-1 and the reverse primer of the qPCR-HRM-2 as reported in Table 1), including the targets of the two qPCR assays, cloned using the TOPO TA Cloning kit for sequencing (Thermo Fisher Scientific, Invitrogen, Waltham, MA, USA). For qPCR-HRM-1, a FPV sequence [FPV strain 1033/2009, (48)] was inserted into the standard plasmid, while for qPCR-HRM-2, an Asian-like CPV-2c sequence (GenBank ID: MW659469) was inserted. The qPCR standard curve was generated by serial ten-fold dilutions of the recombinant plasmids with a known copy number (from 1 × 10^7^ to 1 × 10^1^ copies/μL). These dilutions were tested in triplicate and used as quantification standards to construct a standard curve by plotting the plasmid copy number against the corresponding threshold cycle values. The limit of detection (LOD) for each assay was defined as the lowest plasmid concentration that yielded positive results in all three replicates of each of the three reactions performed. To assess specificity, positive and negative controls were tested. As positive controls, FPV, original CPV-2, different CPV-2 antigenic variant (2a, 2b, 2c) and Asian-like CPV were used. As negative controls, canine and feline pathogens, as well as canine and feline genomic DNA, were included. Specifically, the negative controls comprised canine adenovirus type 1 and 2, *Anaplasma phagocytophilum*, *Leishmania infantum*, feline herpesvirus, *Mycoplasma haemofelis*, *Escherichia coli*, *Proteus mirabilis*, *Pseudomonas aeruginosa*, *Enterobacter cloacae*, *Klebsiella pneumoniae*, *Staphylococcus aureus*, *Enterococcus faecalis*. Intra-assay variability of the two assays was evaluated by testing seven successive 10-fold dilutions (from 1 × 10^1^ to 1 × 10^7^copies/μl) of FPV or Asian-like CPV-2c recombinant plasmid, respectively, in triplicate within the same run. The inter-assay variability was evaluated by testing the same standard plasmid dilutions in triplicate across three different days for each of the two assays. Mean, standard deviation, and coefficient of variation (CV) were calculated. In particular, the CV was calculated as the percentage of the ratio of standard deviation and the mean values were obtained.

### 2.2 Identification and genetic characterization of FPV and CPV

#### 2.2.1 Study population

Dogs and cats diagnosed with FPV or CPV infection at the VTH of the University of Bologna between 2017 and 2023 were included in the study. DNA was extracted from fresh non-stored rectal swabs, feces or blood samples, collected for diagnostic purpose at the time of referring to VTH, using the NucleoSpin Tissue Kit (Macherey-Nagel, Düren, Germany), according to the manufacturer’s instructions, and stored at −20 °C until use. Signalment and clinical data of the dogs and cats included in the study were retrieved from medical records. The study was carried out only on surplus material obtained for diagnostic purposes following owner’s informed consent. No additional sampling was carried out for the purpose of this study and no experimental animals were involved. DNA extracts obtained from the dogs and cats included in the study were tested by the two assays, qPCR-HRM-1 and qPCR-HRM-2, developed in this study. In each run, samples and five 10-fold dilutions of the standard plasmid were repeated in duplicate and a no template control (ultrapure water) was analyzed simultaneously. At the end of the qPCR amplification, the specimens were considered positive if the amplification fluorescence curve increased exponentially, the Tm was specific and the mean of the target copy number obtained from the replicates was greater than the LOD. Differentiation of viruses and variants was carried out on positive specimens by HRM analysis.

#### 2.2.2 Sequencing and bioinformatic analysis

All viruses identified and typed with the developed assays were subsequently sequenced for confirmation and genetic characterization. For this purpose, a previously described end-point PCR (34), amplifying the entire VP2 gene, was carried out using the Phusion Hot Start II High-Fidelity DNA Polymerase Kit (Thermo Fisher Scientific, Life Technologies, Waltham, MA, USA) according to the manufacturer’s instructions. An Asian-like CPV-2c positive sample (GenBank ID: MW659469) used as positive control and a no template control, consisting of ultrapure water, underwent analysis simultaneously. PCR products were visualized under UV after electrophoresis migration on a 1% agarose gel stained with Midori Green Advance DNA Stain (Nippon Genetics, Düren, Germany) in 1X standard tris-acetate-EDTA buffer. Amplicons of the expected size were considered positive, purified using the QIAquick PCR Purification Kit (Qiagen, Hilden, Germany) according to the manufacturer’s instructions and directly sequenced by Sanger method (BioFab Research, Rome, Italy) using both forward and reverse primers. The nucleotide sequences obtained were assembled and aligned with reference sequences available in the GenBank database,^4^ using the ClustalW method implemented in the BioEdit sequence alignment editor version 7.7. The assembled sequences were translated into amino acid sequences using the BioEdit software and specific VP2 residues were analyzed to deduce viral types and antigenic variants (Table 2). Phylogeny of the complete VP2 gene was carried out on nucleotide sequences obtained in this study and reference sequences obtained in the GenBank database with the MEGA 11 software version 11.0.11 (49) using the Maximum-Likelihood method and Tamura 3-parameter model with gamma distribution and invariable sites. One thousand replicates of bootstrap analysis were performed to evaluate the robustness of the phylogenetic tree.

**Table 2:** Amino acid residues in the deduced parvovirus VP2 protein analyzed to deduce viral types and antigenic variants.

Virus	Amino acid residues																	
	5	80	87	93	101	103	267	297	300	305	323	324	370	426	440	555	564	568
FPV	Ala	Lys	Met	Lys	Ile	Val	Phe	Ser	Ala	Asp	Asp	Tyr	Gln	Asn	Thr	Val	Asn	Ala
CPV-2	Ala	Arg	Met	Asn	Ile	Ala	Phe	Ser	Ala	Asp	Asn	Tyr	Gln	Asn	Thr	Val	Ser	Gly
CPV-2a	Ala	Arg	Leu	Asn	Thr	Ala	Phe	Ser/Ala	Gly	Tyr	Asn	Tyr	Gln	Asn	Thr/Ala	Ile/Val	Ser	Gly
CPV-2b	Ala	Arg	Leu	Asn	Thr	Ala	Phe	Ser/Ala	Gly	Tyr	Asn	Tyr/Leu	Gln	Asp	Thr	Val	Ser	Gly
CPV-2c	Ala	Arg	Leu	Asn	Thr	Ala	Phe	Ser/Ala	Gly	Tyr	Asn	Tyr	Gln	Glu	Thr	Val	Ser	Gly
Asian-like CPV-2a	Ala	Arg	Leu	Asn	Thr	Ala	Tyr	Ser/Ala	Gly	Tyr	Asn	Ile	Gln	Asn	Thr/Ala	Val	Ser	Gly
Asian-like CPV-2b	Ala	Arg	Leu	Asn	Thr	Ala	Tyr	Ser/Ala	Gly	Tyr	Asn	Ile	Gln	Asp	Thr/Ala	Val	Ser	Gly
Asian-like CPV-2c	Gly	Arg	Leu	Asn	Thr	Ala	Tyr	Ser/Ala	Gly	Tyr	Asn	Ile	Arg	Glu	Thr	Val	Ser	Gly

Ala, Alanine; Arg, Arginine; Asn, Asparagine; Asp, Aspartic acid; CPV, Canine parvovirus; FPV, Feline panleukopenia virus; Gln, Glutamine; Glu, Glutamic acid; Gly, Glycine; Ile, Isoleucine; Leu, Leucine; Lys, Lysine; Met, Methionine; Phe, Phenylalanine; Ser, Serine; Thr, Threonine; Tyr, Tyrosine; Val, Valine.

## 3 Results

### 3.1 Real-time PCR with high resolution melting assay 1 (qPCR-HRM-1)

**Figure 3:**
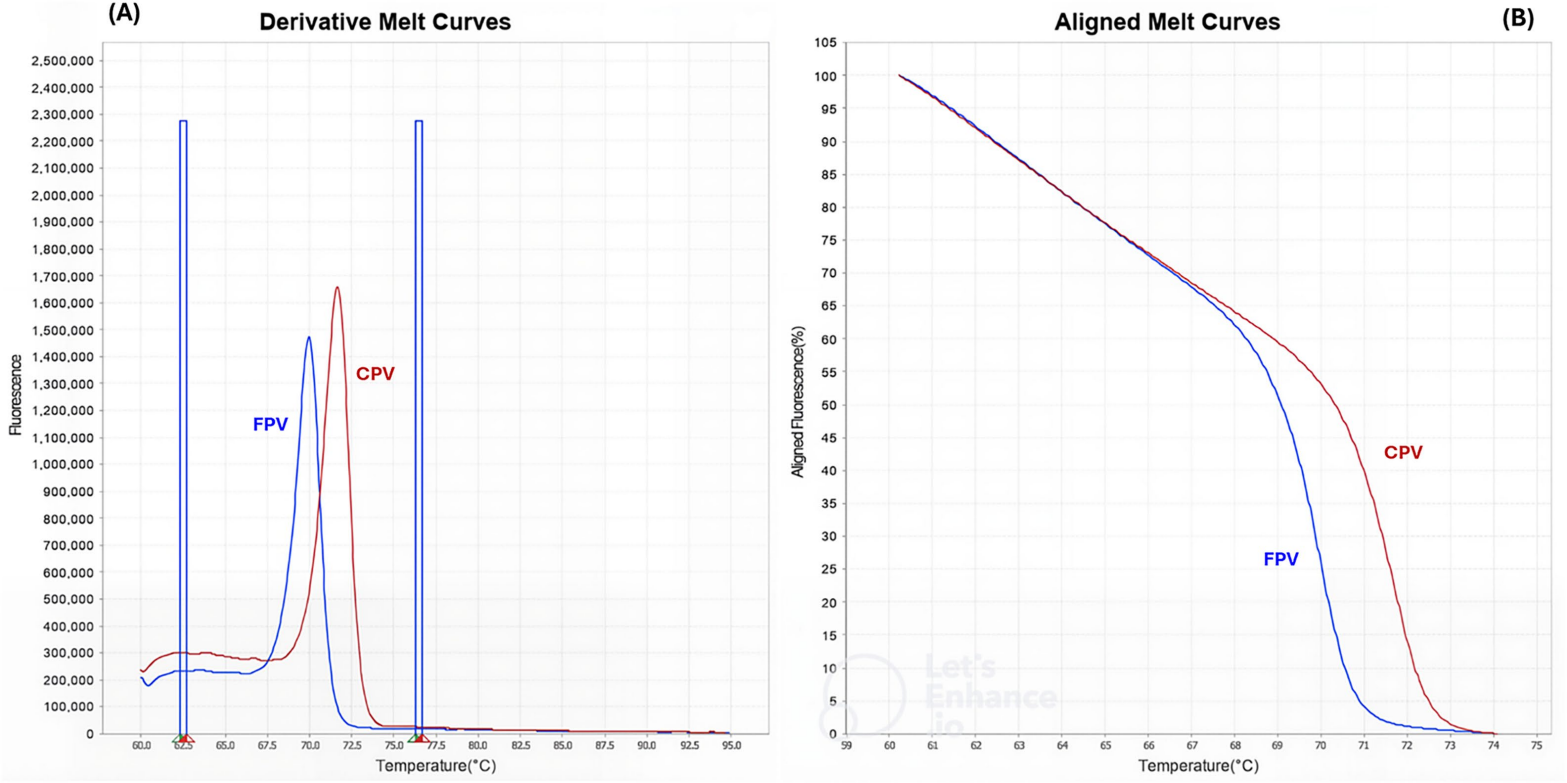
QPCR-HRM-1 analysis. Conventional **(A)** and normalized **(B)** melting curves analysis performed with Applied Biosystems High Resolution Melt Software v3.1 (Thermo Fisher Scientific, Waltham, MA, USA). Two distinct melting profiles were identified: FPV in blue and CPV in red.

**Figure 4:**
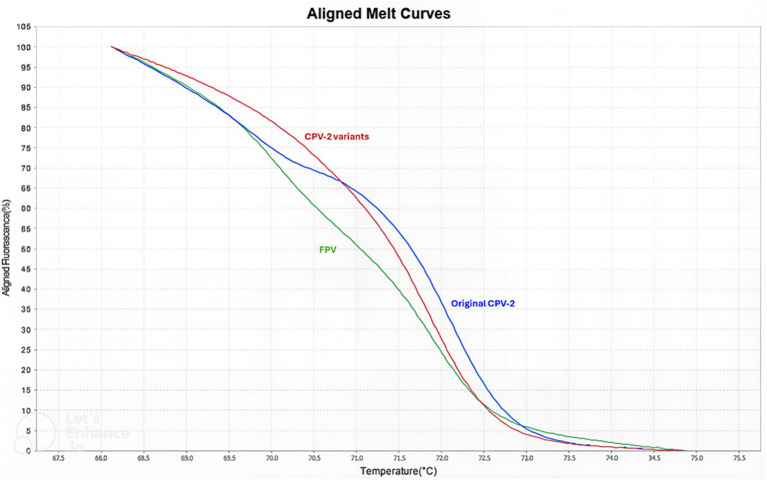
qPCR-HRM-1 analysis performed after heteroduplex formation. Normalized melting curves analysis performed with Applied Biosystems High Resolution Melt Software v3.1 (Thermo Fisher Scientific, Waltham, MA, USA). Three distinct melting profiles were identified: FPV in green, original CPV-2 in blue, and CPV-2 antigenic variants (2a–2c) in red.

The qPCR-HRM-1 assay successfully classified viral DNA into FPV and CPV groups based on melting temperature analysis: 70 °C for FPV and 72 °C for CPV (Figure 3). After heteroduplex formation, HRM analysis was able to discriminate three distinct groups by the formation of specific melting profiles as shown in Figure 4: FPV, original CPV-2 and CPV-2 antigenic variants (2a, 2b, and 2c).

The standard curve was generated by plotting qPCR Ct of each dilution against the known copy numbers of the FPV recombinant plasmid. The resulting slope showed linearity over 7 orders of magnitude ranging from 1 × 10^1^ to 1 × 10^7^ copies/μl showing a high sensitivity of the assay with a LOD of 10 copies/μl. The slope was −3,713 with coefficient of determination (R^2^) > 0.99 and a reaction efficiency (E) of 89.4%. The developed assay successfully detected all species and variants of FPV and CPV. No positive results were obtained from any other pathogen tested. In the intra-assay variability evaluation, a CV of 0.15–5.88 was obtained for all the recombinant plasmid dilutions (Table 3). In the inter-assay variability evaluation, the CV value ranged from 0.98 to 6.92 for all recombinant plasmid dilutions with concentration higher than or equal to 10^2^copies/μl, and of 32.37 for the 1 × 10^1^ plasmid dilution (Table 4), as expected due to Poisson distribution effects, which contribute to greater CV values when quantifying low copy numbers. Despite this increased variability, low viral concentrations were consistently detected across all replicates using this assay.

**Table 3:** Intra-assay variability of the qPCR-HRM assays developed.

qPCR-HRM	Intra-assay variability				
	Samples	Replicate (and assay) numbers	Mean (SD)	Log_10_ mean (SD)	Log_10_ CV%
1	PL7	3 (1)	1.03E+07 (7.05E+05)	7.02 (0.03)	0.42
	PL6	3 (1)	1.19E+06 (1.56E+05)	6.07 (0.06)	0.93
	PL5	3 (1)	1.38E+05 (2.69E+04)	5.13 (0.09)	1.77
	PL4	3 (1)	6.38E+03 (3.27E+02)	3.80 (0.02)	0.58
	PL3	3 (1)	8.36E+02 (8.59E+01)	2.92 (0.004)	0.15
	PL2	3 (1)	8.76E+01 (1.44E+01)	1.94 (0.08)	3.88
	PL1	3 (1)	9.28E+00 (1.17E+00)	0.96 (0.06)	5.88
2	PL7	3 (1)	1.59E+07 (9.72E+05)	7.20 (0.03)	0.37
	PL6	3 (1)	1.19E+06 (1.29E+05)	6.07 (0.05)	0.77
	PL5	3 (1)	7.83E+04 (3.16E+03)	4.89 (0.02)	0.35
	PL4	3 (1)	5.38E+03 (1.81E+03)	3.72 (0.14)	3.74
	PL3	3 (1)	7.97E+02 (4.00E+01)	2.90 (0.02)	0.76
	PL2	3 (1)	8.91E+01 (3.11E+00)	1.95 (0.02)	0.77
	PL1	3 (1)	2.01E+01 (1.62E+01)	1.22 (0.32)	26.39

qPCR-HRM = Real-time PCR with High-Resolution Melting analysis; PL* = recombinant plasmid dilution.

**Table 4:** Inter-assay variability of the qPCR-HRM assays developed.

qPCR-HRM	Inter-assay variability				
	Samples	Replicate (and assay) numbers	Mean (SD)	Log_10_ mean (SD)	Log_10_ CV%
**1**	PL7	3 (3)	3.09E+07 (2.0E+07)	7.39 (0.28)	3.92
	PL6	3 (3)	1.35E+06 (3.14E+05)	6.03 (0.06)	0.98
	PL5	3 (3)	7.39E+04 (5.56E+04)	4.76 (0.33)	6.92
	PL4	3 (3)	5.03E+03 (1.95E+03)	3.65 (0.21)	5.82
	PL3	3 (3)	1.16E+03 (4.77E+02)	3.03 (0.17)	5.53
	PL2	3 (3)	1.13E+02 (2.47E+01)	2.02 (0.07)	3.47
	PL1	3 (3)	1.24E+01 (8.57E+00)	1.02 (0.33)	32.37
**2**	PL7	3 (3)	1.46E+07 (2.59E+06)	7.16 (0.08)	1.13
	PL6	3 (3)	1.06E+06 (1.15E+05)	6.02 (0.04)	0.75
	PL5	3 (3)	7.98E+04 (8.00E+03)	4.89 (0.05)	0.93
	PL4	3 (3)	6.45E+03 (1.40E+03)	3.79 (0.09)	2.40
	PL3	3 (3)	9.39E+02 (1.89E+02)	2.96 (0.08)	2.85
	PL2	3 (3)	1.20E+02 (3.54E+01)	2.06 (0.12)	5.95
	PL1	3 (3)	1.46E+01 (4.79E+00)	1.08 (0.12)	11.24

qPCR-HRM = Real-time PCR with High-Resolution Melting analysis; PL* = plasmid dilution.

### 3.2 Real-time PCR with high resolution melting assay 2 (qPCR-HRM-2)

**Figure 5:**
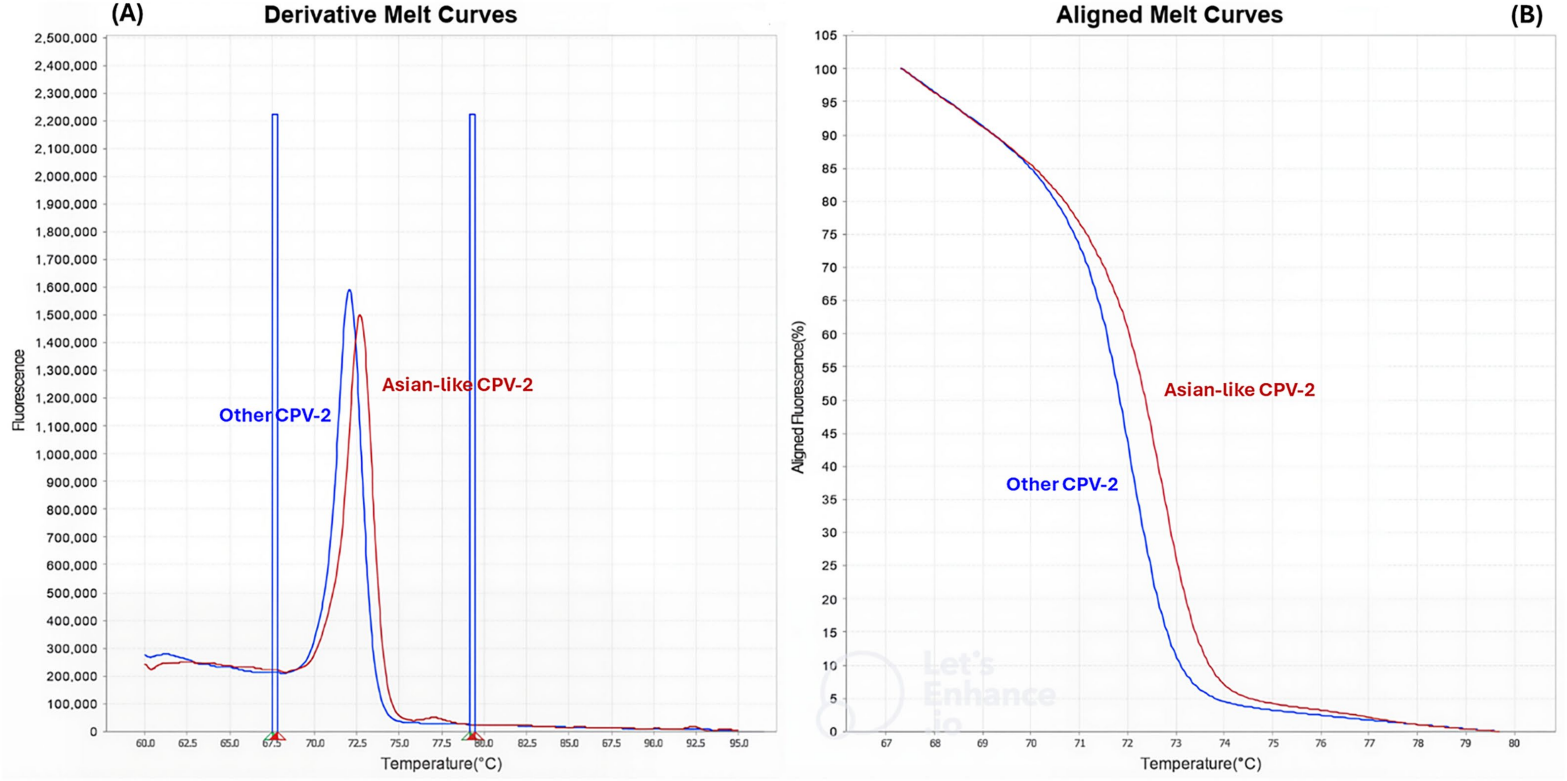
qPCR-HRM-2 analysis. Conventional **(A)** and normalized **(B)** melting curves analysis performed with Applied Biosystems High Resolution Melt Software v3.1 (Thermo Fisher Scientific, Waltham, MA, USA). Two distinct melting profiles were identified: Asian-like CPV-2 in red and other CPVs in blue.

The qPCR-HRM-2 assay successfully classified viral DNA in two groups based on melting temperature analysis: 73 °C for Asian-like CPV-2 and 72 °C for other CPVs (Figure 5).

The standard curve was generated by plotting qPCR Ct of each dilution against the known copy numbers of the Asian-like CPV-2c recombinant plasmid. The resulting slope showed linearity over 7 orders of magnitude ranging from 1 × 10^1^ to 1 × 10^7^ copies/μl showing a high sensitivity of the assay with a LOD of 10 copies/μl. The slope was −3,378 with coefficient of determination (R^2^) > 0.99 and a reaction efficiency (E) of 97.7%. The developed assay successfully detected all CPV variants. No positive results were obtained from any other pathogen tested. In the intra-assay variability evaluation, a CV of 0.35–3.74 was obtained for all the recombinant plasmid dilutions with concentration higher than or equal to 10^2^copies/μl, and of 26.39 for the 1 × 10^1^ plasmid dilution (Table 3). In the inter-assay variability evaluation, the CV value ranged from 0.75 to 5.95 for all recombinant plasmid dilutions with concentrations higher than or equal to 10^2^copies/μl, and of 11.24 for the 1 × 10^1^ plasmid dilution (Table 4). As for qPCR-HRM-1, increased CV variability for low target concentrations was expected due to Poisson distribution effects, but, despite this, low viral concentrations were consistently detected across all replicates using this assay.

### 3.3 Identification and genetic characterization of FPV and CPV

Thirty-three animals were included in the study, including 21/33 (64%) dogs and 12/33 (36%) cats. The dog population consisted in 10/21 (48%) females and 11/21 (52%) males with a median age of 4 months (1 month - 5 years and 9 months). The cat population was composed of the same number of females and males (6/12, 50%) with a median age of 6 months (1 month–3 years and 7 months). For each animal just one sample was tested, specifically, rectal swabs or feces from 31/33 (93.9%) animals and blood from 2/33 (6.1%) animals. Two viruses identified in blood sample of two dogs had been sequenced in a previous study conducted at the same facility [Lab ID 636/2019 GenBank ID OM892823 and Lab ID 637/2019 GenBank ID OM892824, (50)].

All animals and samples tested positive with the two molecular assays developed. Using the qPCR-HRM-1 the viral DNA detected in 11/12 (91.7%) cats grouped as FPV, in 2/21 (9.5%) dogs grouped as original CPV-2, and in 20 animals, 19/21 (90.5%) dogs and 1/12 (8.3%) cat, grouped as CPV-2 antigenic variants 2a, 2b or 2c. The qPCR-HRM-2, carried out on the 22 animals (21 dogs and 1 cat) tested positive for CPV DNA with the previous assay, allowed to differentiate the obtained amplicons in two groups. Specifically, the viral DNA detected in 10/21 (47.6%) dogs were classified as Asian-like CPV and 12 animals (11/21, 52.4% dogs and 1/12, 8.3% cat) were classified as other CPVs.

**Figure 6:**
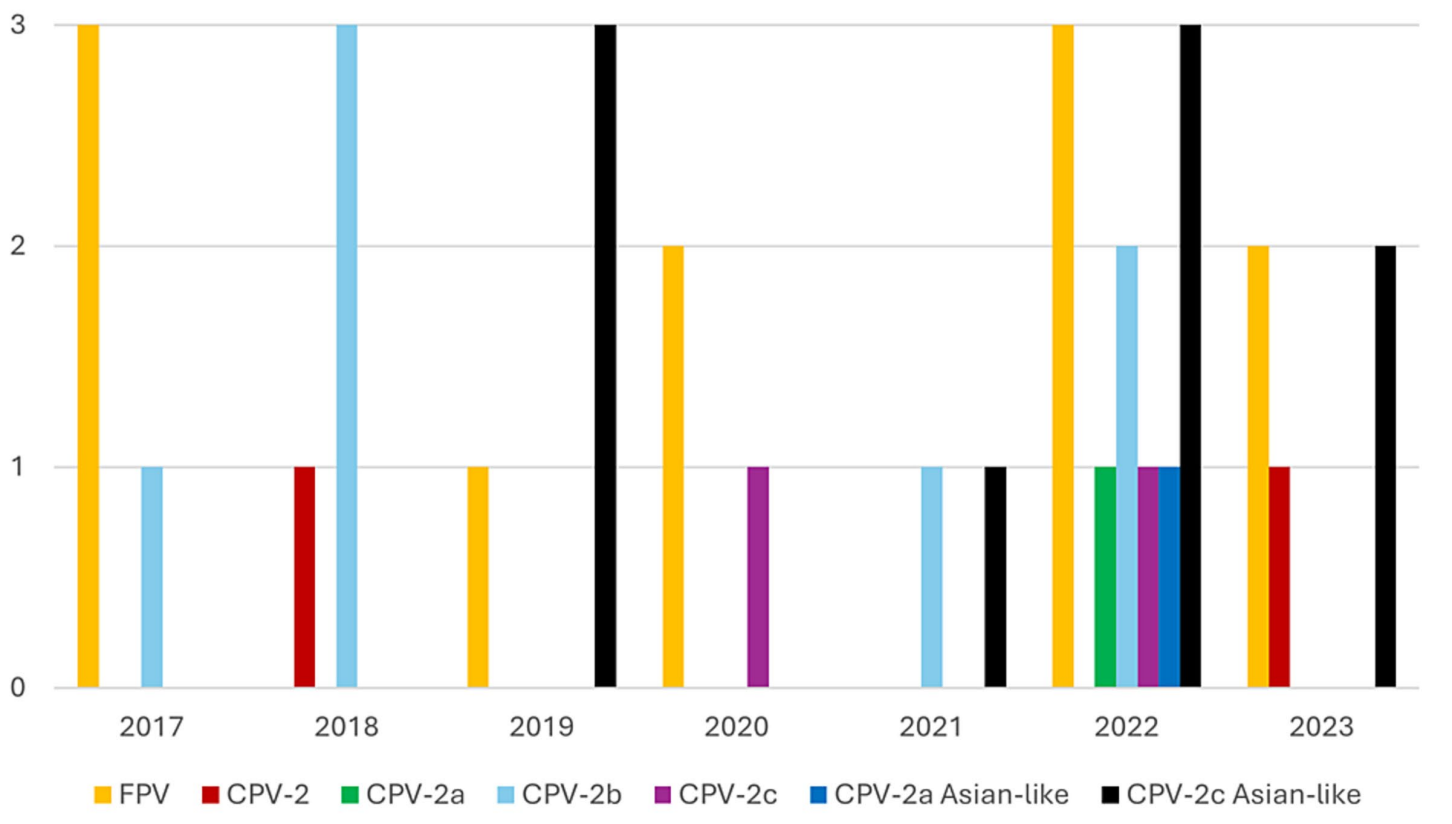
Distribution of each antigenic variant through the years of the study. *x*-coordinate: year of sampling; *y*-coordinate: number of animals.

After complete VP2 gene sequencing (1755 nts), the identified viruses were classified based on specific amino acid residues. Specifically, 11/33 (33.3%) viruses were FPV, 2/33 (6.1%) were original CPV-2, 2/33 (6.1%) were CPV-2a (including one Asian-like CPV-2a), 7/33 (21.2%) were CPV-2b (not including Asian-like CPV-2b) and 11/33 (33.3%) were CPV-2c (including nine Asian-like CPV-2c). The distribution of each antigenic variant through the years of sampling is shown in Figure 6. The viral classification performed with the two qPCR-HRM assays was confirmed for all animals and samples tested, as reported in Table 5.

**Table 5:** Comparison of viral classification obtained by qPCR-HRM assays and sequencing results.

Sample ID	Host	qPCR-HRM-1	qPCR-HRM-2	Sequencing
39/2017	Cat	FPV		FPV
68/2017	Cat	FPV		FPV
1299/2017	Cat	FPV		FPV
1000/2019	Cat	FPV		FPV
779/2020	Cat	FPV		FPV
1158/2020	Cat	FPV		FPV
941/2022	Cat	FPV		FPV
259/2022	Cat	FPV		FPV
1923/2022	Cat	FPV		FPV
608/2023	Cat	FPV		FPV
1225/2023	Cat	FPV		FPV
242/2018	Dog	Original CPV-2	Other CPVs	Original CPV-2
416/2023	Dog	Original CPV-2	Other CPVs	Original CPV-2
1476/2022	Dog	CPV-2a/2b/2c	Other CPVs	CPV-2a
136/2018	Dog	CPV-2a/2b/2c	Other CPVs	CPV-2b
281/2018	Dog	CPV-2a/2b/2c	Other CPVs	CPV-2b*
283/2018	Dog	CPV-2a/2b/2c	Other CPVs	CPV-2b*
1536/2021	Dog	CPV-2a/2b/2c	Other CPVs	CPV-2b*
13/2022	Dog	CPV-2a/2b/2c	Other CPVs	CPV-2b*
745/2022	Dog	CPV-2a/2b/2c	Other CPVs	CPV-2b*
10/2017	Cat	CPV-2a/2b/2c	Other CPVs	CPV-2b*
210/2020	Dog	CPV-2a/2b/2c	Other CPVs	CPV-2c
973/2022	Dog	CPV-2a/2b/2c	Other CPVs	CPV-2c
400/2022	Dog	CPV-2a/2b/2c	Asian-like CPV	Asian-like CPV-2a
276/2019	Dog	CPV-2a/2b/2c	Asian-like CPV	Asian-like CPV-2c
636/2019	Dog	CPV-2a/2b/2c	Asian-like CPV	Asian-like CPV-2c
637/2019	Dog	CPV-2a/2b/2c	Asian-like CPV	Asian-like CPV-2c
1438/2021	Dog	CPV-2a/2b/2c	Asian-like CPV	Asian-like CPV-2c
1170/2022	Dog	CPV-2a/2b/2c	Asian-like CPV	Asian-like CPV-2c
1228/2022	Dog	CPV-2a/2b/2c	Asian-like CPV	Asian-like CPV-2c
1868/2022	Dog	CPV-2a/2b/2c	Asian-like CPV	Asian-like CPV-2c
1725/2023	Dog	CPV-2a/2b/2c	Asian-like CPV	Asian-like CPV-2c
1888/2023	Dog	CPV-2a/2b/2c	Asian-like CPV	Asian-like CPV-2c

CPV, Canine parvovirus; FPV, Feline panleukopenia virus; qPCR-HRM, Real-time PCR with High-Resolution Melting analysis. * CPV-2b with distinctive amino acid residues 371Gly and 418Thr. Column qPCR-HRM-1: in blue FPV; in green original CPV-2: in red CPV-2 antigenic variants. Column qPCR-HRM-2: in yellow other CPVs; in orange Asian-like CPVs. Column sequencing: in blue FPV; in green original CPV-2: in purple CPV-2 antigenic variants; in orange Asian-like CPVs.

**Figure 7:**
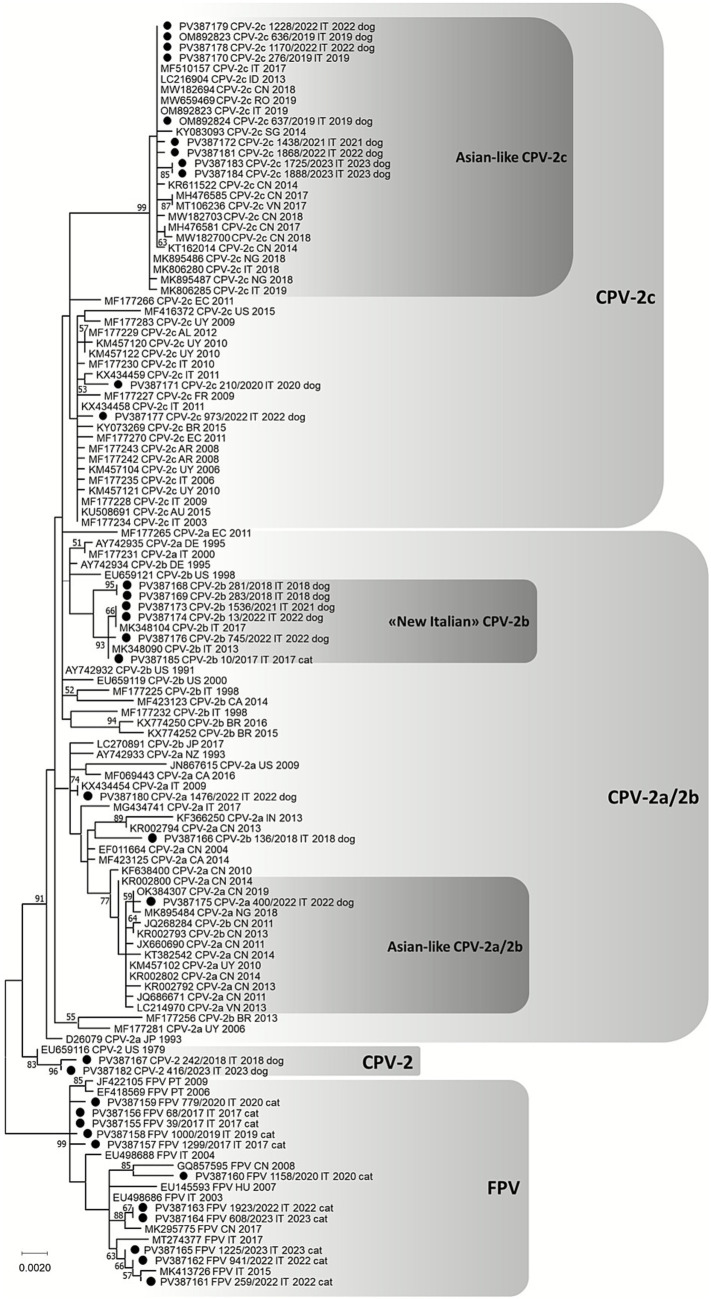
Nucleotide phylogenetic tree constructed on the complete VP2 gene of FPV and CPV (1755 nts) using maximum likelihood method and Tamura 3-parameter model with gamma distribution and invariable sites implemented in MEGA 11 software version 11.0.11. One thousand replicates of bootstrap analysis were performed to evaluate robustness of the phylogenetic tree, and bootstrap values ≥ 50% are indicated on respective branch. The reference sequences are labeled as follows: GenBank ID, virus, country of origin (AL, Albania; AR, Argentina; AU, Australia; BR, Brazil; CA, Canada; CN, China; DE, Germany; EC, Ecuador; FR, France; HU, Hungary; ID, Indonesia; IN, India; IT, Italy; JP, Japan; NG, Nigeria; NZ, New Zealand; PT, Portugal; RO, Romania; SG, Singapore; US, United States; UY, Uruguay; VN, Vietnam), date of collection (or submission date). ● = sequences obtained in this study labeled as follows: GenBank ID, virus, Lab ID, country of origin, date of collection, host. New Italian CPV-2b: CPV-2b with distinctive amino acid mutations Ala371Gly and Ile418Thr.

The nucleotide identity between FPV sequences obtained in this study ranged from 99.4 to 100% and was 99.2–100% with reference ones. At the amino acid level, all FPV identified in this study were identical, except for the FPV identified in cat 1299/2017 (GenBank ID: PV387157), which exhibited a Ser13Pro substitution, and the FPV identified in cat 1158/2020 (GenBank ID: PV387160), which displayed the two amino acid substitutions Ile232Val and Leu562Val. The phylogenetic analysis revealed that the FPV identified in this study grouped with viruses identified worldwide and FPV 1158/2020 clustered with a virus identified in China in 2008 (GenBank ID: GQ857595, Figure 7).

The nucleotide identity of CPV sequences obtained in this study ranged from 98.5 to 100% with each other and with reference sequences. The two CPV-2a sequences obtained in this study showed a nucleotide identity of 99.6%. The CPV-2a identified in dog 1476/2022 (GenBank ID: PV387180) was identical to a virus detected in Italy in 2009 (GenBank ID: KX434454), whereas the CPV-2a identified in dog 400/2022 (GenBank ID: PV387175) had amino acid residues compatible with Asian-like viruses and grouped strictly with other viral strains from China and Nigeria (Figure 7).

In contrast, the CPV-2b sequences obtained in this study showed higher genetic diversity, with nucleotide identity ranging from 99.3 to 100%. Six CPV-2b (Lab ID: 10/2017 GenBank ID: PV387185; Lab ID: 281/2018 GenBank ID: PV387168; Lab ID: 283/2018 GenBank ID: PV387169; Lab ID: 1536/2021 GenBank ID: PV387173; Lab ID: 13/2022 GenBank ID: PV387174; Lab ID: 745/2022 GenBank ID: PV387176) exhibited the distinctive amino acid residues 371Gly and 418Thr, previously reported in the literature for viruses detected in Italy and tentatively named as “New Italian” CPV-2b. They phylogenetically grouped with other “New Italian” CPV-2b (Figure 7) and were highly conserved at the amino acid level, with only a single substitution (Pro13Ser) observed in two viruses (Lab ID: 281/2018 GenBank ID: PV387168 and Lab ID: 283/2018 GenBank ID: PV387169). The other CPV-2b identified in dog 136/2018 (GenBank ID: PV387166) differed from all reference sequences available in GenBank database, exhibited the distinctive amino acid residues 324Ile, and it appeared phylogenetically more closely related to CPV-2a strains from Asia (Figure 7).

The CPV-2c sequences obtained in this study showed a nucleotide identity ranging from 98.9 to 100%. Two CPV-2c identified in dogs 210/2020 (GenBank ID: PV387171) and 973/2022 (GenBank ID: PV387177) differed from each other and were closely phylogenetically related to viruses circulating in Italy for the longest time (Figure 7). In particular, the CPV-2c 973/2022 had the two distinctive amino acid residues 219Ile and 384Gln. The other nine CPV-2c (Lab ID: 276/2019 GenBank ID: PV387170; Lab ID: 636/2019 GenBank ID: OM892823; Lab ID: 637/2019 GenBank ID: OM892824; Lab ID: 1438/2021 GenBank ID: PV387172; Lab ID: 1170/2022 GenBank ID: PV387178; Lab ID: 1228/2022 GenBank ID: PV387179; Lab ID: 1868/2022 GenBank ID: PV387181; Lab ID: 1725/2023 GenBank ID: PV387183; Lab ID: 1888/2023 GenBank ID: PV387184) were mostly identical and had amino acid residues compatible with Asian-like viruses, with which they phylogenetically grouped (Figure 7). Only the Asian-like CPV-2c 1868/2023 (GenBank ID: PV387181) exhibited a distinctive amino acid substitution (Ile164Val).

Furthermore, two original CPV-2 sequences were obtained from two dogs (Lab ID: 210/2020 GenBank ID: PV387171 and Lab ID: 416/2023 GenBank ID: PV387182, Figure 7), which showed a nucleotide identity of 99.8% and exhibited the amino acid residues 219Val, 375Asn and 386Lys.

## 4 Discussion

In this study, two qPCR assays were developed and validated for the detection and genetic characterization of FPV and CPV infecting dogs and cats through HRM analysis. These assays enable both a sensitive and specific diagnosis, useful for clinical treatment and patient management, and a rapid epidemiological assessment of circulating strains.

The first qPCR-HRM-1 assay detects and differentiates FPV from original CPV-2 and CPV-2 antigenic variants (2a, 2b, and 2c), providing an initial characterization. It determines whether a positive result is due to the detection of a vaccine (original CPV-2) or field (FPV or CPV-2 antigenic variants) viral strain. The second qPCR-HRM-2 assay differentiates Asian-like CPV-2 strains from other CPVs. These strains, detected in recent years but likely circulating since the early 2000s in Asia (51), have been reported multiple times in Europe and Africa (31–34). The pathogenic potential of Asian-like CPVs remain unknown, but their prevalence appears to be increasing, suggesting higher transmissibility and fitness in hosts (29, 30). Both assays showed great sensitivity with a limit of detection of 10 DNA viral copies/μl and an excellent specificity, confirming their potential application for diagnostic purpose.

To validate the reliability of the developed assays on clinical samples and perform an epidemiological investigation on the viruses circulating in Northern Italy, 33 dogs and cats previously tested positive for parvovirus DNA between 2017 and 2023, were analyzed. The median age of the tested animals was approximately four and 6 months for dogs and cats respectively, with a marked frequency of cases in animals under 1 year of age. This aligns with literature data indicating that the period of highest risk of infection is between weaning and 6 months of age (15, 52, 53). This is attributed primarily to the tropism of the virus for mitotically active cells, which are more prevalent in growing animals. Additionally, maternal antibody titers decline around 6–8 weeks of age, making puppies susceptible to infection until completion of the vaccination schedule at 16 weeks of age (54). In adults, infection, also observed in this study, was predominantly associated with incomplete or absent vaccination. Therefore, although the study population was relatively small in number, it provides a representative picture of the typical clinical presentation of parvovirus infection. The characterization of the viruses sequenced in this study also enables to assess FPV and CPV strains evolution in Italy and update previously conducted epidemiological studies in the country (5, 40, 55–60).

In the present study, FPV was detected in 11/12 (91.7%) cats and not in dogs, confirming the host specificity of this virus (61). The high values of nucleotide identity calculated on the complete VP2 gene between FPV identified in this study and reference strains confirms the low variability of this virus (5, 55, 62). However, the identified FPV exhibited nucleotide mutations which, although synonymous, highlight genetic heterogeneity among circulating viruses. This heterogeneity is further supported by phylogeny, which does not reveal a unique grouping for detected FPV. The Ser13Pro substitution identified in yhe virus detected in cat 1299/2017 has never been reported in FPV strains but has been observed in several CPV-2 strains, without any association with specific pathogenicity patterns (63). Indeed, residue 13 is located in the internal N-terminal region of the capsid and is not associated with exposed antigenic loops. Therefore, substitutions at this position are likely to have a limited impact on antigenicity, although they may be relevant for capsid assembly or stability (21). In contrast, the mutations identified in the virus detected in cat 1158/2020 had been previously reported. Specifically, the Ile232Val substitution was identified in several cat viruses from China by Tang and colleagues, who suggested that it may represent a novel pattern of VP2 genetic evolution in FPV strains, even though the potential functional consequences of this mutation remain unknown (64). Residue 232 has been suggested to contribute to cross-species viral transmission among carnivores, due to its putative involvement in receptor-binding regions (65). The Leu562Val substitution has also been previously reported in FPV strains, without any proposed biological function (66). Although residue 562 has not been identified as a key host-range determinant, its proximity to VP2 residues 564 and 568 (positions implicated in host-range differences between FPV- and CPV-like viruses) suggests that the Leu562Val substitution could potentially modulate local capsid conformation and thereby indirectly contribute to host-range phenotypes. This hypothesis is supported by the study of Allison and colleagues, who identified a mutation at amino acid 562 as potentially linked to the viral host adaptation (67).

CPV VP2 gene sequences showed higher variability compared to FPV, confirming a continuous evolution of this virus (68). CPV-2a was detected in only two dogs and not in cats. CPV-2a was reported as the predominant variant infecting dogs in Italy (63, 69), but in recent years a progressive increase in the circulation of CPV-2b and CPV-2c in dogs has been reported, to the detriment of this antigenic variant (40, 70), as confirmed by our results. Furthermore, the two CPV-2a identified in this study were detected in 2022, the year with the higher sample size, potentially increasing the likelihood of detecting this variant. Differently, CPV-2b was detected in six dogs and one cat. In the early 2000s, the detection of CPV-2b in dogs from Italy progressively decreased over time (18, 40), only to start increasing again with the spread of viruses characterized by the distinctive VP2 amino acid residues 371Gly and 418Thr, and tentatively named as “New Italian” CPV-2b (57, 59, 63, 70, 71). Six out of the seven CPV-2b detected in this study, including the one identified in the cat, had these distinctive amino acid residues, suggesting an increased fitness for the “New Italian” CPV-2b and supporting a progressive replacement of the old CPV-2b. Additionally, four of these viruses carried Serine in the VP2 amino acid residue 13, firstly reported by Battilani and colleagues (63). CPV-2b had already been associated to cat disease (64, 72), but this is the first report of “New Italian” CPV-2b infection in cats. This finding suggest that “New Italian” CPV-2b does not entail changes in host range compared with the already known CPV-2 variants and could cause infection in cats with clinical implications, the severity of which will need to be assessed. The virus identified in 136/2018, despite belonging to CPV-2b variant, appeared to be more closely related phylogenetically to CPV-2a and presented an amino acid profile intermediate with Asian-like CPV viruses. This highlights the continuous evolution of CPV and also emphasizes how its classification into the antigenic variants 2a, 2b, and 2c, although widely adopted, is limiting and arbitrary, as already stated by several authors (57, 73–75).

CPV-2c was the most frequently identified CPV variant, detected in eleven dogs (52.4% of the dogs tested). These data confirm the trend of increasing diffusion of this antigenic variant in Italy, already reported by different authors (63, 70). Notably, nine CPV-2c and one CPV-2a, for a total of 10/22 (45.4%) CPV identified in this study, had amino acid residues in the deduced VP2 protein characteristic of Asian-like viruses. Specifically, the nine CPV-2c were characterized by 5Gly, 267Tyr, 324Ile, and 370Arg residues, previously reported in Europe (Italy and Romania), Africa (Nigeria and Egypt) and Asia (31–34, 76–78), and the CPV-2a displayed the distinctive amino acid profile 267Tyr, 324Ile, and 440Ala first described by Zhou and colleagues (29) in Asia. Interestingly, the CPV-2c strain in dog 1868/2023 showed an Ile164Val substitution. This mutation was previously identified in a virus detected in an Italian grey wolf (79), however no information regarding its biological implications is reported. This amino acid appears usually highly conserved, suggesting that the substitution identified in this study may reflect genetic drift or local adaptation (80). In general, the Asian-like viruses identified in this study appear to be highly conserved at both nucleotide and amino acid levels. Moreover, they are closely related to strains identified in Asia, suggesting an origin linked to an initial importation and subsequent diffusion. In contrast, the other CPV-2a, 2b and 2c viruses identified in this study exhibited genetic heterogeneity and showed higher similarity to strains previously reported in Italy. This supports the hypothesis that these viruses are autochthonous and that the observed mutations resulted from the continuous evolution rather than introduction from other countries.

In two dog puppies aged two and 4 months, who had received a dose of vaccine in the weeks preceding diagnosis (1 and 10 weeks, respectively), two original CPV-2 were identified, supporting a potential vaccine origin. These two viruses were not genetically identical and exhibited the VP2 amino acid residues 219Val and 386Lys, differently from the American reference strain type 2 [GenBank ID: EU659116, (81)]. These substitutions were introduced to attenuate a specific live-virus vaccine strain and patented by the manufacturer (82) but were also reported in vaccine strains before patent submission (62). The amino acid residues 219Val and 386Lys were found in CPV-2 strains identified in unvaccinated dogs from Tanzania, suggesting a potential natural transmission of vaccine-derived viruses (83) and were also interestingly found in a CPV-2c identified in this study (Lab ID: 973/2022 GenBank ID: PV387177). Additionally, these two potentials vaccine-derived CPV-2 harbored the Asp375Asn substitution in the deduced VP2 protein, a mutation previously reported for CPV-2b strains (25). This finding may support the emergence of mutations in vaccine strains during replication in the host, with effects that should be further investigated.

The two qPCR-HRM assays successfully classified all the clinical samples tested, confirming their potential use as diagnostic technique. Indeed, despite the increasing cost per single reaction compared with end-point PCR and non-HRM qPCR, these assays result in a substantial cost saving when gene sequencing is required. Combined with faster turnaround times and higher sensitivity, these features make them a valid and efficient alternative.

This study has some limitations. The first and foremost is the detection limit of the developed methods, which, although sensitive, could still be improved to diagnose cases of persistent infections usually characterized by low viral titers. To correctly discriminate original CPV-2 and CPV-2 antigenic variants, the qPCR-HRM-1 requires an additional step (heteroduplex formation); this procedure adds complexity and elongated the analysis, but it still remains convenient compared to sequencing. Furthermore, the two qPCR-HRM assays should be associated with at least another assay to discriminate the CPV-2a, 2b, and 2c antigenic variants. Another limitation of this study is the small size of the study population, which may compromise the reliability of the epidemiological study, not allowing the generalization of the data to the entire national territory and restricting it to areas surrounding the VTH, specifically in Northern Italy.

In conclusion, the real-time PCR assays associated with High-Resolution Melting analysis developed and validated in this study correctly detected and classified all the FPV and CPV tested, highlighting their reliability and usefulness for both diagnostic and epidemiological purposes. The genetic characterization of viruses circulating in Northern Italy over the seven-year study period confirmed a higher prevalence of FPV in cats and CPV-2c in dogs, consistent with previous reports. Moreover, the analysis highlighted a predominant presence of newly emerging variants (Asian-like CPV and “New Italian” CPV-2b) which exhibited a high degree of similarity among the viruses belonging to them. Overall, this study confirms the extensive nucleotide heterogeneity of the VP2 gene in CPV, driven by ongoing viral evolution, whereas FPV remains relatively stable over time. The biological implications of most mutations remain unclear, although some are widely detected, while others occur sporadically, highlighting the need for continued surveillance of parvovirus evolution to monitor epidemiological trends and potential pathogenetic shifts.

## Data Availability

The datasets generated for this study can be found in the AMSActa UNIBO repository at https://doi.org/10.6092/unibo/amsacta/8271. The nucleotide sequences generated and analyzed during the current study are available in GenBank Database, accession numbers: PV387155-PV387185.

## Footnotes

1 http://www.ncbi.nlm.nih.gov/genbank, accessed on 01/11/2024

2 http://blast.ncbi.nlm.nih.gov/Blast.cgi, accessed on 05/11/2024

3 http://www.basic.northwestern.edu/biotools/OligoCalc.html, accessed on 05/11/2024

4 https://www.ncbi.nlm.nih.gov/nucleotide/, accessed on 11/02/2025
